# Prospective Evaluation of a Commercial Dengue NS1 Antigen Rapid Diagnostic Test in New Caledonia

**DOI:** 10.3390/microorganisms10020346

**Published:** 2022-02-02

**Authors:** Enagnon Kazali Alidjinou, Sylvie Tardieu, Isabelle Vrenken, Didier Hober, Ann-Claire Gourinat

**Affiliations:** 1 Laboratoire de Virologie ULR3610, University of Lille, CHU Lille, F-59000 Lille, France; didier.hober@chru-lille.fr; 2 Microbiology Laboratory, Centre Hospitalier Territorial de Nouvelle-Calédonie, 98835 Dumbea, France; ann-claire.gourinat@cht.nc; 3 Laboratory Department, Centre Hospitalier du Nord, 98860 Kone, France; sylvie.tardieu@chn.nc (S.T.); isabelle.vrenken@chn.nc (I.V.)

**Keywords:** dengue virus, NS1 antigen, rapid diagnostic test, RT-PCR, evaluation

## Abstract

Dengue virus infection is endemic in New Caledonia, with outbreaks occurring every year. We evaluated the Biosynex^®^ Dengue NS1 antigen rapid diagnostic test (RDT) for the early diagnosis of dengue in patients attending a local hospital in northern New Caledonia. Samples collected from patients suspected of dengue infection were tested with RDT at the local laboratory, and then sent to the reference laboratory for confirmation with real-time RT-PCR. A total of 472 samples were included during the study period. RT-PCR yielded a positive result in 154 samples (32.6%). The sensitivity and specificity of the NS1 antigen RDT were 79.9% and 96.2%, respectively. The performance of the RDT varied by the time of sampling and dengue virus serotype. In conclusion, Biosynex^®^ Dengue NS1 antigen RDT showed a sensitivity and a specificity in the upper range usually reported for this type of test. Several factors can lead to a suboptimal sensitivity, and negative samples with suggestive clinical features should be retested with reference methods.

## 1. Introduction

Dengue is the most rapidly spreading mosquito-borne viral disease and is currently endemic in more than 100 countries worldwide [1]. The global burden of dengue is estimated around 400 million infections per year, 96 million of which are symptomatic [2]. 

In New Caledonia, dengue virus circulation has increased since 2008, causing recurrent outbreaks, with cases detected every year. About 7266 cases were reported during the 2016–2018 period [3].

The clinical features during the early phase of dengue infection are non-specific, and it is challenging to differentiate it from the other causes of acute febrile illness. Therefore, laboratory diagnosis is essential to distinguish dengue from other diseases causing similar clinical presentation [4]. According to the CDC, during the acute phase of dengue (up to 7 days after symptom onset), laboratory confirmation is made in a single diagnostic specimen by detecting viral genomic sequences with RT-PCR or the dengue nonstructural protein 1 (NS1) antigen by immunoassay. IgM antibody testing can allow identification of additional infections in this period (4–7 days after fever onset), but is most useful in the convalescent phase (>7 days after fever onset) when the previous tests can be negative, although NS1 has been reported to be positive up to 12 days after fever onset [5]. 

Nucleic acid amplification tests (NAAT) are not widely available since they require sophisticated equipment and well-trained staff. Therefore, NS1 antigen detection represents an alternative for the early diagnosis of dengue, especially in less equipped laboratories [6]. 

NS1 antigen detection is one of the most important developments in dengue diagnostics in the last decade. The NS1 antigen is present in blood during both primary and secondary infections. Therefore, the major commercial diagnostics manufacturers have early developed ELISA-format tests, and then RDT-based NS1 antigen tests [7]. RDTs that meet the ASSURED criteria are indeed needed in settings with limited laboratory diagnostic resources [8]. RDTs are cost-effective and easy to perform, with results usually available in less than 30 min. These tests provide the opportunity for point-of-care diagnosis, and can also be used for screening during outbreaks in remote healthcare centers [7,9].

Many studies have evaluated the most common NS1 antigen RDTs, using either ELISA or NAAT as a reference, and have reported various performance data depending on the time of collection, the dengue serotype, and the type of infection (primary or secondary) [7,9]. 

In this study, we report an independent prospective evaluation of the Biosynex^®^ Dengue NS1 antigen RDT, a recent commercial NS1 antigen RDT, for the early diagnosis of dengue infection.

## 2. Materials and Methods

### 2.1. Setting and Study Design

New Caledonia is an archipelago in the South Pacific, located approximately 1200 km east of Australia and 1500 km northwest of New Zealand. New Caledonia has a land area of 18,576 km^2^ (7172 sq mi), with a population of 268,767 inhabitants in 2014 (http://www.isee.nc, accessed 11 September 2019). 

Located in Dumbea (southern New Caledonia), the “Centre Hospitalier Territorial” is the largest hospital, and the hospital laboratory is the reference for most specialized diagnostic investigations. 

For the diagnosis of dengue virus infection, the tools available include RT-PCR and dengue serology, which are usually implemented based on the time of sampling and the clinical picture. At the hospital of Kone, located in northern New Caledonia, a screening in patients with dengue-like illness is usually performed using an RDT, before the sample is sent to the reference laboratory for confirmation and virus typing.

This study included samples tested at the hospital of Kone (northern New Caledonia) using a NS1 antigen RDT (Biosynex Dengue NS1 assay), and sent for confirmation from March 2017 to December 2018. Only the samples obtained between 0 and 7 days after symptoms onset, which underwent both NS1 antigen and RT-PCR, were included in this evaluation. The staff was blind to the RDT results at the time of testing in the reference laboratory.

### 2.2. Laboratory Methods

#### 2.2.1. Dengue NS1 Antigen RDT

The Biosynex^®^Dengue NS1 assay (Biosynex SA, Illkirch-Graffenstaden, France is a lateral flow assay for the rapid detection of the NS1 antigen. The test was performed in accordance with the manufacturer’s instructions. Briefly, 100 µL of serum was transferred into the sample area. After a migration time of 15 min, the presence of two colored lines in the results window (control and patient) indicated a positive result. An initial reading was completed by the technician and then checked by the medical biologist. A weak line in the patient window was considered positive.

#### 2.2.2. Dengue RT-PCR and Virus Typing

A taqman one-step RT-PCR was performed using previously described primers and probes [10]. Briefly, nucleic acids were extracted from 200 μL of serum specimen by using the MagNA Pure LC 2.0 instrument with the MagNA Pure LC Nucleic Acid Isolation Kit- High Performance (Roche Diagnostics, Meylan, France), according to the manufacturer’s instructions. The amplification was run either on the QuantStudio 6 Flex Real-Time PCR System or the 7500 Real-Time PCR System (Thermo Fisher Scientific, Illkirch-Graffenstaden, France). For positive samples, dengue virus typing was performed on the same extract using a fourplex real-time reverse transcriptase PCR assay, as previously described [11].

### 2.3. Statistical Analysis

Descriptive statistics were performed using Excel software (Microsoft, Issy-les-Moulineaux, France). Agreement, sensitivity, specificity, positive predictive value (PPV), and negative predictive value (NPV) were determined according to the Clinical and Laboratory Standards Institute’s guidelines, [12] using RT-PCR as a gold standard. Confidence interval (CI) values were calculated, using the QuickCalcs tool (GraphPad, San Diego, CA, USA). Fischer exact test was used for comparisons, and a *p* value < 0.05 was statistically significant.

### 2.4. Ethical Statement

This study was based on medical records, in compliance with the French reference methodology MR-004, established by French National Commission on Informatics and Liberties (CNIL), and approved by the Institutional data protection authority of CHT New Caledonia.

## 3. Results

### 3.1. Patients and Samples

**Figure 1:**
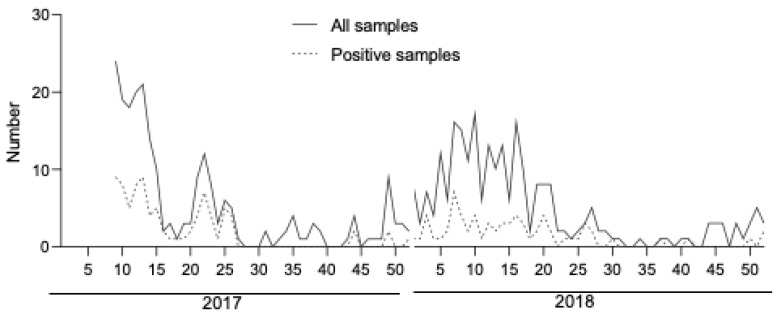
Weekly distribution of samples. A weekly distribution of all samples (solid line) and RT-PCR positive samples (dashed line) included during the study period is shown.

A total of 472 samples were included in this study. The median age of patients was 26.9 years old (range between 0 and 94 years old), and they were mainly female (52.1%). The median time of sampling (TOS) was 2 days post fever onset (IQR, 1 and 4 days). The repartition of samples according to the TOS is shown in Table 1. A positive result with RT-PCR was found in 154 samples (32.6%). No association was found between positivity and age (*p* = 0.4) or sex (*p* = 0.3). The weekly distribution of samples is shown in Figure 1. The distribution pattern shows that cases occurred throughout the year, during both the cool season (from mid-May to mid-November) and the hot season (from mid-November to mid-May). However, the great majority of cases (70.1%) were detected during the hot season. Dengue virus typing was performed in 151 samples. Dengue virus 1 (DENV-1) was the most common serotype (68.2%), followed by DENV-2 (27.2%) and DENV-3 (3.9%). No DENV-4 was identified during the study period (See Table 1).

### 3.2. Overall RDT Performance

The overall agreement between NS1 antigen RDT and RT-PCR was 90.9%. The overall sensitivity of the NS1 antigen RDT compared to RT-PCR was 79.9% (CI95 = 72.8 to 85.5), with a specificity of 96.2% (CI95 = 93.5 to 97.9). The PPV was 91.1% (CI95 = 85 to 95), while the NPV was 90.8% (CI95 = 87.2 to 93.5) (See Table 2).

### 3.3. RDT Performance Varied by Time of Sampling and Dengue Serotype

The agreement between NS1 antigen RDT and RT-PCR was significantly better for samples obtained between 0 and 4 days post onset than for those collected between 5 and 7 days post onset (92.5% versus 80.3%, *p* = 0.0065). The sensitivity and specificity of RDT for the first sample group was 80.7% (CI95 = 73.2 to 86.6) and 98.2% (CI95 = 95.7 to 99.4), respectively. RDT was less performant for the second sample group, with a sensitivity of 73.7% (CI95 = 50.9 to 88.6) and a specificity of 83.3% (CI95 = 69.1 to 92) (Table 2).

The performance of RDT was also dependent on dengue serotype. A sensitivity of 92.2% (CI95 = 85.2 to 96.2) was found for DENV-1, while it decreased to 50% (CI95 = 35.5 to 64.5) for DENV-2. Regarding DENV-3 positive samples, RDT yielded a positive result in 5 out of 6 samples (estimated sensitivity at 83.3%) (Table 2).

## 4. Discussion

The evaluation of diagnostic accuracy of dengue NS1 RDTs is important in endemic areas such as New Caledonia, where these tools are extensively used. To choose a RDT for routine diagnosis, it can be very difficult to directly compare the diagnostic performance of two different tests evaluated in two different studies. Multiple evaluation studies of different commercially available RDTs in different field conditions are essential to figure out the performance of each test.

In this study, we prospectively assessed, for the first time, the performance of the Biosynex^®^ Dengue NS1 antigen for the routine diagnosis of dengue in New Caledonia. Both NS1 and IgM/IgG RDTs are marketed by the manufacturer, but the present study only evaluated the NS1 antigen test, as compared to the “in-house” RT-PCR. 

Overall, a good performance was found for the Biosynex^®^ NS1 RDT, with a sensitivity of 79.9% and a specificity of 96.2%. The RDT performance was better in very early samples (0–4 days post fever onset). In addition, the test was more sensitive in detection of DENV-1 positive samples.

The most common commercially available NS1 antigen RDTs include the Dengue NS1 Strip (Bio-Rad, Marnes-la-Coquette, France), the Panbio^®^ Dengue Early Rapid Test (Standard Diagnostics, Suwon, South Korea), the SD Bioline Dengue Duo (Standard Diagnostics), and the CTK Biotech Dengue NS1 antigen test, (CTK Biotech, San Diego, CA, USA) [9].

Several reports in the literature have evaluated dengue NS1 RDTs. In 2012, a review by Blacksell, including the Dengue NS1 Strip by Bio-Rad (12 studies), SD Bioline Dengue Duo by Standard Diagnostics (4 studies,) and the Dengue Early Rapid Test by Panbio^®^ (3 studies), showed a considerable range of sensitivities among the DENV RDTs evaluated (48.5% to 98.9%), although specificities were reasonably consistent (90.6–100%). The performance was dependent on the test used, the geographic area, the reference comparator, or the time of sampling [7].

Similarly, a WHO collaborative work by Hunsperger et al. found a highly variable performance with a sensitivity ranging from 38% to 71% during primary infection, and from 21% to 55% during secondary infection [13]. A large multicentric evaluation (four countries) of the SD Bioline Dengue Duo found a sensitivity ranging from 49.7% to 92.9%, and a specificity from 22.2% to 89.9% depending on the country, the serotype, and the time of sample collection [14].

Data from significant recent evaluation studies [15,16,17,18,19,20,21,22,23], using RT-PCR as reference, are summarized in Table 3. These data confirm the variable performance observed in earlier studies. As shown in Table 3, the performance of a given RDT (e.g., SD Bioline) can significantly change between two similar studies. 

This variability can be explained by the high impact of the duration of illness before sample collection, which can be subjective information.

Another factor is the serotype distribution across studies. A lower sensitivity was found in this study for DENV-2, in agreement with previous reports that showed low detection rates for DENV-2 and DENV-4 [24,25].

It was previously shown that the combination of DENV NS1 and the IgM antibody test can improve the diagnostic performance [26], but in this study, we have only assessed NS1 RDT, because our objective was the diagnosis of dengue infection in acute phase.

To the best of our knowledge, only one recent retrospective study reported data on the Biosynex dengue NS1 RDT [15]. The performance observed for the Biosynex RDT in our study is very similar to that reported in the previously published study. More interestingly, in that study, the Biosynex RDThad the best sensitivity among the five evaluated RDTs.

The strength of our study relies on the population sample size and the prospective design, which results in a more realistic diagnostic performance. This study is limited by the lack of possible distinction between primary and secondary infections. Previous studies have shown that NS1 antigen detection was more sensitive in dengue primary infections compared to secondary infections [21,27]. However, data in the literature are conflicting regarding the impact of the type of infection (primary or secondary) on RDT sensitivity [22].

In conclusion, the results of this first evaluation of a dengue NS1 RDT in New Caledonia agree with findings from other reports that have generally shown that NS1 RDTs have high specificity, but there are several factors that influence the sensitivity, including the time of sampling and dengue serotype. Therefore, negative samples with suggestive clinical features should be retested with reference methods.

## Figures and Tables

**Table 1:** Sample description.

Characteristics	Number	Percentage
Time of sampling (*n* = 472)		
0–4 days	411	87.1
5–7 days	61	12.9
RT-PCR result (*n* = 472)		
Negative	318	67.4
Positive	154	32.6
Dengue virus serotype		
DENV-1	103	66.9
DENV-2	42	27.2
DENV-3	6	3.9
DENV-4	0	0

**Table 2:** RDT performance.

Biosynex Dengue NS1 Ag RDT	Reference (RT-PCR)	
RDT Overall performance	Positive	Negative
Positive	123	13
Negative	31	306
Sensitivity (95% CI)	79.9% (72.8–85.5%)	
Specificity (95% CI)	96.2% (93.5–97.9%)	
Positive predictive value (95% CI)	91.1% (85.0–95.0%)	
Negative predictive value (95% CI)	90.8% (87.2–93.5%)	
RDT Performance by time of sampling		
<5 days		
Sensitivity (95% CI)	80.7% (73.2–86.6%)	
Specificity (95% CI)	98.2% (95.7–99.4%)	
≥5 days		
Sensitivity (95% CI)	73.7% (50.9–88.6%)	
Specificity (95% CI)	83.3% (69.1–92.0%)	
RDT Sensitivity by Dengue serotype		
Dengue 1 Sensitivity (95% CI)	92.2% (85.2–96.2%)	
Dengue 2 Sensitivity (95% CI)	50.0% (35.5–64.5%)	
Dengue 3 Sensitivity (95% CI)	83.3% (41.8–98.9%)	

**Table 3:** Recent studies evaluating the performance of dengue NS1 Ag RDTs using RT-PCR as reference.

Study	Country	Number of Samples	Assay Name (Details Below *)	Sensitivity	Specificity	Comments
[15]	Indonesia	149	Biosynex ^1^	80%	100%	Samples were collected within 5 days post symptom onset. Significant number of DENV-1, 2, 3, and 4 positive samples. Sensitivity ranged from 44.4 to 100% across dengue serotypes for RDTs. All RDTs performed better for DENV-3.
			CTK Biotech ^2^	71%	100%	
			Boson Biotech ^3^	73%	100%	
			Panbio ^4^	74%	100%	
			SD Bioline ^5^	73%	100%	
This study	New caledonia	471	Biosynex ^1^	79.9%	96.2%	Samples were collected within 7 days post symptom onset. Significant number of DENV-1 and 2 positive samples. The sensitivity was 92.2 and 50% for DENV-1 and DENV-2, respectively.
[17]	Taiwan	173	NS1 Ag Bio-Rad ^6^	85.3%	94.6%	Samples were collected within 6 days post symptom onset. Significant number of DENV-1, 2, and 3 positive samples. Sensitivity ranged from 84 to 100% across dengue serotypes for RDTs.
			CTK Biotech ^2^	89%	73%	
			SD Bioline ^5^	89.7%	91.9%	
[16]	Solomon Islands	412	SD Bioline ^5^	90.9%	100%	Samples collected within 6 days post symptom onset. Data were not available for sensitivity across dengue serotypes.
			CTK Biotech ^2^	92.6%	78.8%	
[18]	India	253	Dengue DAY 1 ^8^	91.1	93.6	Samples were collected within 5 days post symptom onset. Significant number of DENV-1, 2, and 3 positive samples. The sensitivity was higher than 90% for DENV-1, 2, and 3.
[19]	Malaysia	490	ViroTrack ^7^	62.3%	95%	Samples were collected within 11 days post symptom onset. Significant number of DENV-1, 2, and 3 positive samples. Sensitivity ranged from 54.8 to 96.7% across dengue serotypes for RDTs. Both NS1 RDTs performed better for DENV-3 (sensitivity > 90%) than DENV-1 and 2.
			SD Bioline ^5^	52.4%	97.7%	
[20]	Thailand	778(433 patients)	ViroTrack ^7^	85.5%	97%	Samples collected within 5 days post symptom onset. Significant number of DENV-1, 2, 3, and 4 positive samples. Sensitivity ranged from 75 to 90% across dengue serotypes for both RDTs.
			SD Bioline ^5^	78.9%	99%	
[21]	Myanmar	172 (children)	Humasis Dengue ^9^	63.3%	100%	Samples collected within 3–7 days post symptom onset. Significant number of DENV-1 and 2 positive samples. Sensitivity from 52 to 88% across dengue serotypes for RDTs.
			SD Bioline ^5^	48.6%	100%	
			CareUS Dengue ^10^	79.8%	100%	
[22]	Brazil	500	SD Bioline ^5^	38.6%	98.2%	The median duration of illness before sample collection was 2 (IQR: 2–4) days. Significant number of DENV-1, 2, and 4 positive samples. The sensitivity was 55.6%, 46%, and 61.2% for DENV-1, DENV-2 and DENV-4, respectively. (DENV typing not performed on all positive samples).
[23]	Myanmar	202	CareUS Dengue ^10^	72.1%	87.1%	Samples were collected within 7 days post symptom onset. Data were not available for sensitivity across dengue serotypes.
			Humasis Dengue ^9^	68.6%	90.3%	
			Wondfo Dengue ^11^	67.1%	91.9%	

* Dengue rapid diagnostic tests evaluated, ^1^ Dengue NS1 BSS (Biosynex SA, Illkirch-Graffenstaden, France), ^2^ Answer Dengue Ag Rapid Test (CTK Biotech, San Diego, CA, USA), ^3^ Rapid Dengue NS1 Antigen Test Card (Xiamen Boson Biotech, China), ^4^ Panbio Dengue Early Rapid (Standard Diagnostics, Suwon, South Korea), ^5^ SD Bioline Dengue NS1 Ag (Standard Diagnostics, Suwon, South Korea), ^6^ Dengue NS1 Ag Strip (Bio-Rad, Marnes-la-Coquette, France), ^7^ ViroTrack Dengue Acute (BluSense Diagnostics, København, Denmark), ^8^ Dengue DAY 1 Test” diagnostic kit (J. Mitra & Co., New Delhi, India), ^9^ Humasis Dengue NS1 (Launch Diagnostics, Longfield, UK), ^10^ CareUS Dengue NS1 (WellsBio, Seoul, Korea), ^11^ Wondfo Dengue Combo Kit, (Biotech, Guangzhou, China).

## Data Availability

The data presented in this study are available on request from the corresponding author.
